# Adolescent Changes in Cellular Proliferation in the Dentate Gyrus of Male and Female C57BL/6N Mice Are Resilient to Chronic Oral Corticosterone Treatments

**DOI:** 10.3389/fnbeh.2018.00192

**Published:** 2018-08-24

**Authors:** Ashna Shome, Razia Sultana, Alina Siddiqui, Russell D. Romeo

**Affiliations:** Department of Psychology and Neuroscience and Behavior Program, Barnard College of Columbia University, New York City, NY, United States

**Keywords:** adolescence, corticosterone, puberty, oral administration, proliferation, neurogenesis, hippocampus

## Abstract

Adolescent development is marked by significant changes in neurobiological structure and function. One such change is the substantial adolescent-related decline in cellular proliferation and neurogenesis in the dentate gyrus of the hippocampal formation. Though the behavioral implications of these developmental shifts in cell proliferation are unclear, these changes might contribute to the altered cognitive and emotional functions associated with puberty and adolescence. The significant decrease in cellular proliferation throughout adolescence might make the hippocampus more vulnerable to perturbations during this developmental stage, particularly to factors known to disrupt neurogenesis, such as chronic exposure to stress-related hormones. To examine this possibility, we first measured cellular proliferation in the dentate gyrus of male and female C57BL/6N mice before and after adolescence and then assessed both cellular proliferation and the number of immature neurons in mice treated with oral corticosterone for 4 weeks during either adolescence or adulthood. We found significant age-related decreases in hippocampal cellular proliferation in both males and females. Though the greatest decrease in proliferation was during adolescence, we also observed that proliferation continued to decline through young adulthood. Despite the significant effect of chronic oral corticosterone on body weight gain in both the adolescent- and adult-treated males and females and the subtle, but significant suppressive effect of corticosterone on the number of immature neurons in the adolescent-treated males, cell proliferation in the hippocampus was unaffected by these treatments. These data show that the substantial adolescent-related change in cellular proliferation in the dentate gyrus is largely unaffected by chronic oral corticosterone exposure in males and females. Thus, despite being vulnerable to the metabolic effects of these chronic corticosterone treatments, these results indicate that the developmental changes in cellular proliferation in the dentate gyrus are relatively resilient to these treatments in mice.

## Introduction

Adolescence is associated with significant neurobiological changes, including substantial declines in cellular proliferation and neurogenesis in the dentate gyrus of the hippocampal formation in both rats and mice (Heine et al., 2004; Kim et al., 2004; He and Crews, 2007; Hodes et al., 2009; Ho et al., 2012). Though the hippocampal formation plays a role in many cognitive and emotional processes (Fanselow and Dong, 2010), the neurobehavioral implications of these adolescent changes in proliferation are unclear. In addition to development, exposure to stress and stress-related hormones, such as corticosterone, alter cell proliferation and neurogenesis in the dentate gyrus (Schoenfeld and Gould, 2012; Opendak and Gould, 2015). Given the marked increase in stress-related physiological and behavioral dysfunctions associated with adolescence, ranging from obesity to mood disorders (Turner and Lloyd, 2004; Dahl and Gunnar, 2009; Lee et al., 2014; Poyrazoglu et al., 2014), perturbations of adolescent hippocampal development by stress-related hormones might contribute to the change in these vulnerabilities.

We have recently shown that chronically exposing adolescent and adult male mice to oral corticosterone leads to significant changes in body weight and adiposity at both ages, but the trajectory and magnitude of these metabolic changes are different before and after adolescence (Kinlein et al., 2017). In particular, despite similarly elevated circulating levels of corticosterone achieved by these treatments, oral corticosterone during adolescence results in initial reduced weight gain followed by increases in body weight, while in adulthood these treatments lead to more linear and substantial increases in both body weight and adiposity (Kinlein et al., 2017). The impact of chronic oral corticosterone treatments on hippocampal cellular proliferation and neurogenesis during adolescence is currently unknown, but may also show age-dependent effects like those observed in the context of metabolism. As alluded to above, it has been shown that exposing adult rats and mice to chronically elevated corticosterone levels reduce hippocampal proliferation and neurogenesis (Murray et al., 2008; David et al., 2009; Brummelte and Galea, 2010; Rainer et al., 2012; Kott et al., 2016). Thus, given the substantial developmental change in proliferation and neurogenesis and the ability of corticosterone to disrupt these processes, it is possible that the chronic oral corticosterone treatments known to affect metabolism differentially during adolescence and adulthood will also result in age-dependent perturbations of these neurobiological parameters.

The purpose of the present set of experiments was to further explore adolescent-related changes in hippocampal proliferation and determine the effects of chronic oral corticosterone on hippocampal proliferation and number of immature neurons in both adolescent and adult male and female mice. Specifically, in the first set of experiments, we examined changes in hippocampal cellular proliferation in male and female mice before and after adolescence, as well during young adulthood. Based on studies in male mice (He and Crews, 2007), we hypothesized that female mice would also show adolescent-related decreases in hippocampal proliferation. In the second set of experiments, we exposed male and female mice to oral corticosterone treatments during either adolescence or adulthood. Given the greater change in hippocampal cellular proliferation and neurogenesis during adolescence (Heine et al., 2004; Kim et al., 2004; He and Crews, 2007; Hodes et al., 2009; Ho et al., 2012), the age-dependent sensitivity to oral corticosterone in the context of metabolism, and the effects of corticosterone on these parameters in adulthood (Murray et al., 2008; David et al., 2009; Brummelte and Galea, 2010; Rainer et al., 2012; Kott et al., 2016), we hypothesized that corticosterone treatment would lead to different effects in the adolescent- compared to adult-treated mice. Finally, though we did not compare males and females directly, the inclusion of both sexes in these studies allowed us to explore whether males and females are affected differently by these treatments, as previous studies report sex differences in the response of the hippocampus to stress-related hormones (Gobinath et al., 2015).

## Materials and Methods

### Animals and Housing

Male and female C57BL/6N mice were obtained from Charles River Laboratories (Wilmington, MA, USA) and allowed to acclimate for at least 1 week prior to the start of the experiments. Mice were housed in pairs (same sex and age) in polycarbonate cages (28 × 17 × 12 cm) with bed-o’cobs 1/4 inch bedding and maintained on a 12-h light dark schedule (lights on at 8:00 h). The temperature was maintained at 21 ± 2°C and mice had *ad libitum* access to water and rodent chow (Lab Diet #5012; PMI Nutrition International LLC, Brentwood, MO, USA). The stage of the estrous cycle was not determined in the female mice. All procedures were approved by the Institutional Animal Care and Use Committee of Columbia University.

### Experimental Designs and Tissue Collections

**Figure 1:**
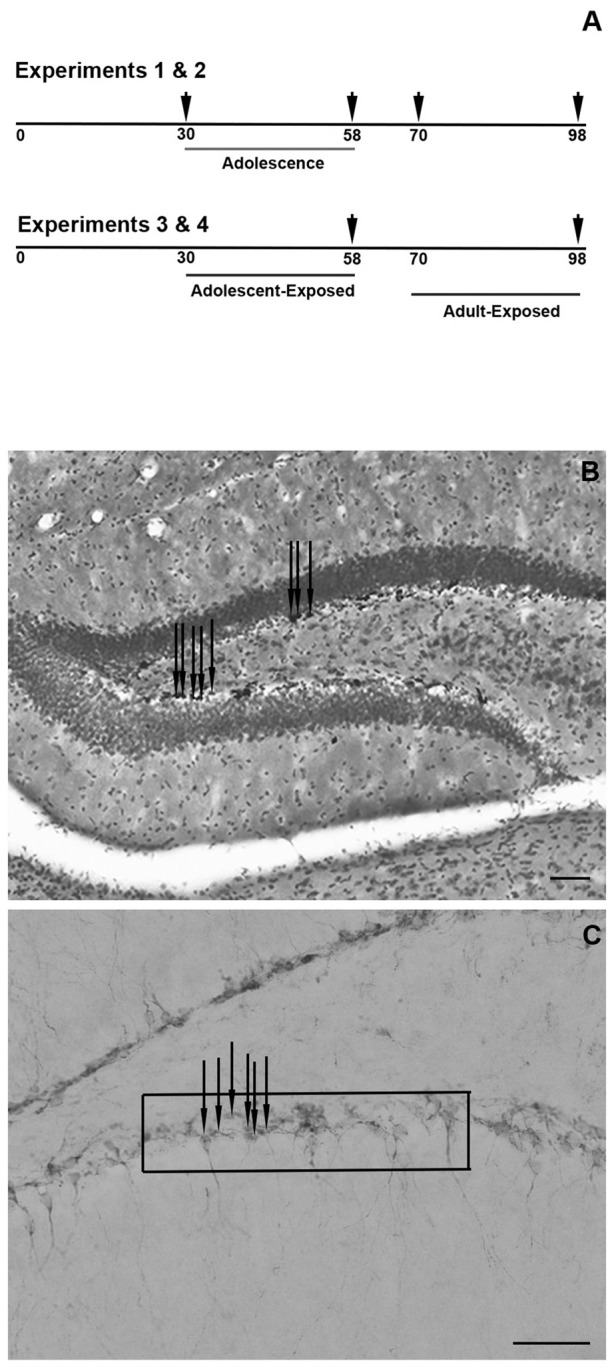
Schematic timeline of the experiments, with arrowheads indicating the times of tissue collections **(A)**. Representative photomicrograph of the counter-stained dentate gyrus and Ki-67-positive cells **(B)** and representative photomicrograph of the doublecortin (DCX)-positive cells in the dentate gyrus and the approximate placement of the grid used to quantify the cells **(C)** Scale bars in panels **(B,C)** = 50 μm. Note that the arrows in panels **(B,C)** are indicating a few examples of Ki-67- and DCX-positive cells, respectively.

Four experiments were conducted (Figure 1A). In the first two experiments, untreated male (Experiment 1) and female (Experiment 2) mice were weighed and perfused (see below) at 30, 58, 70, or 98 days of age (d) and brains were collected (*n* = 4–6 per age). Though the exact age span that defines adolescence and young adulthood in mice is unclear, these ages are operationally defined in these experiments as pre-adolescent (30 days), post-adolescent (58 days), young adult (70 days) and adult (98 days). In the second two experiments, pre-adolescent (30 days) and young adult (70 days) males (Experiment 3) and females (Experiment 4) were exposed to one of two treatments: 0 or 100 μg/ml corticosterone (C2505; Sigma-Aldrich, St. Louis, MO, USA) in a 1% ethanol and tap water vehicle (*n* = 6–8 per age and dose). The dose and vehicle used in these studies were based on previously published experiments in adolescent and adult mice (Kinlein et al., 2017). As corticosterone is hydrophobic, it was first dissolved in 100% ethanol via sonication and then added to tap water to a 1% concentration. Animals were exposed to these treatments for 4 weeks, and therefore, ended at either 58 days or 98 days. Treatments were terminated ~1 h prior to tissue collection. A pilot study revealed that exposing adolescent or adult mice to the 1% ethanol vehicle for 4 weeks did not significantly affect the number of proliferating cells or immature neurons in the dentate gyrus, and thus, to reduce animal numbers, a tap water only control group was not included in these experiments (Romeo, unpublished observation). Moreover, as tissues from males and females were collected from different sex-specific cohorts of animals and processed at different times, results from each sex were analyzed separately.

For all experiments, animals were perfused after being weighed and administered an overdose of ketamine (80 mg/kg, i.p.p) and xylazine (5 mg/kg, i.p.). Transcardial perfusions were conducted using heparinized saline followed by 4% paraformaldehyde in 0.1 M phosphate buffer (PB). Brains were removed and post-fixed in 4% paraformaldehyde for 24 h and then incubated in 20% sucrose in 0.1 M PB for 24 h. Brains were snap frozen on powdered dry ice and stored at −80°C until they were sectioned at 35 μm on a coronal plane. The sections were stored in cryoprotectant (1:1 of 20% sucrose in 0.1 M PB and ethylene glycol) at −20°C until immunohistochemistry was performed.

### Immunohistochemistry

For all the experiments, 3–4 anatomically matched sections through the dorsal hippocampus separated by 105 μm (corresponding to plates 43–47 in a standard mouse atlas; Franklin and Paxinos, 2008), were processed for either Ki-67 to measure cellular proliferation or doublecortin (DCX) to measure the number of immature neurons. The sections were washed in 0.1 M PB followed by a 5 min incubation in 0.3% H_2_O_2_ and washed with 0.1 M PB with 0.1% Triton-X-100 (PBT). Sections were then incubated for 1 h in 2% normal goat serum (NGS), and then in either rabbit anti-Ki-67 (1:8,000; AB15580; Abcam, Cambridge, MA, USA) or guinea pig anti-DCX (1:10,000, AB2253; Millipore Sigma, Burlington, MA, USA) for 24 h at 4°C. Sections were then washed in PBT and incubated in goat anti-rabbit or goat anti-guinea pig secondary (1:200; Vector Laboratories, Burlingame, CA, USA) and then exposed to Avidin-Biotin Complex (1:250; Vectastain ABC Kit, Vector Laboratories) for 1 h at room temperature. The tissue was then washed in 0.1 M phosphate buffer saline (PBS) and exposed to 3,3′diaminobenzidine (DAB) in a 3 M sodium acetate buffer containing 0.05% H_2_O_2_ for 5 min followed by washes in PBS. For Ki-67, the DAB was nickel-enhanced. The tissue was mounted on Fisher Brand Plus slides (Fischer Scientific, Pittsburg, PA, USA) dried and exposed to 70%, 95% and 100% ethanol, followed by xylenes, and cover slipped with DPX (06552, Sigma-Aldrich). Tissue processed for Ki-67 was counter-stained with cresyl violet (C1791, Sigma-Aldrich) prior to coverslipping to measure the cross-sectional area of the dentate gyrus (see below).

### Microscopy and Histological Quantification

Ki-67-positive cells in both the upper and lower blades of the dentate gyrus were counted using a light microscope with a 10× objective (Zeiss 200 M Axiovert), while the cross-sectional area of the dentate gyrus was analyzed with ImageJ from pictures taken under a 2.5× objective (Figure 1B). Bilateral assessments were made from each section and number of cells and cross-sectional areas were averaged. Based on the cross-sectional measurements, Ki-67-positive cells are expressed as the average number of cells per 100 μm^2^ of dentate gyrus. Figure 1B provides a representative photomicrograph of the Ki-67 cell counts and cross-sectional area of the dentate gyrus.

DCX-positive cell counts were made under a 40× objective in both the upper and lower blades of the dentate gyrus by placing a grid of 10,000 μm^2^ superimposed on top of the images (Figure 1C). Bilateral counts from each stained section were averaged and data are expressed as the average number of DCX-positive cells per 10,000 μm^2^. Figure 1C provides a representative photomicrograph of DCX-positive cells and the approximate placement of the grid used for analysis.

### Statistical Analyses

Prior to statistical analyses, the normality of the data sets was confirmed with Shapiro-Wilk normality tests. In Experiments 1 and 2, one-way ANOVAs were used to analyze differences in body weights and the number of Ki-67-positive cells and cross-sectional areas of the dentate gyrus at 30 days, 58 days, 70 days and 98 days. In Experiments 3 and 4, two-way ANOVAs (age of exposure × treatment condition) were used to analyze the number of Ki-67- and DCX-positive cells and cross-sectional area of the dentate gyrus in response to either 0 or 100 μg/ml of oral corticosterone exposure during 4 weeks of either adolescence or adulthood. Significant main effects and interactions were further analyzed with Tukey’s honestly significant difference *post hoc* tests. Data are reported as the mean ± SEM and differences were considered significant at *p* < 0.05. All statistical analyses were performed using GraphPad PRISM, version 7.04 (GraphPad Software Inc., San Diego, CA, USA).

## Results

### Experiments 1 and 2: Developmental Changes in Hippocampal Cellular Proliferation

#### Experiment 1

**Figure 2:**
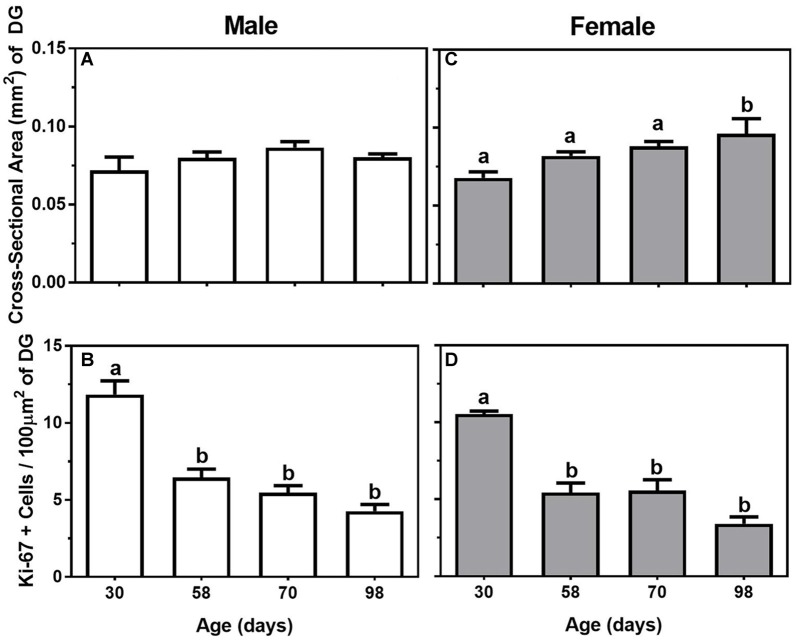
Mean (±SEM) cross-sectional area (mm^2^) of the dentate gyrus (DG) and number of Ki-67-positive cells per 100 μm^2^ of DG in 30-, 58-, 70-, and 98-day-old male (**A,B**; left panels) and female (**C,D**; right panels) mice. Bars that share a letter are not significantly different from one another.

In males, body weight increased significantly during adolescence and young adulthood (*F*_(3,20)_ = 69.02, *P* < 0.05), such that 98-day males weighed the most while the 30-day males weighed the least (Table 1). In the hippocampus, though there was no significant effect of age on the cross-sectional area of the dentate gyrus (*P* = 0.24; Figure 2A), the number of Ki-67-positive cells per 100 μm^2^ of dentate gyrus decreased significantly during adolescence (*F*_(3,20)_ = 28.50, *P* < 0.05). Specifically, 30-day males had significantly greater numbers of proliferating cells than 58-day, 70-day, or 98-day males (Figure 2B). Though not statistically significant, there appears to be a trend toward a continued decrease in the number of Ki-67-positive cells through 58-day, 70-day and 98-day animals.

**Table 1:** Mean (±SEM) body weight of male and female mice in Experiments 1 (male mice) and 2 (female mice).

Sex and days of age (d)	Body weight (g)
Male 30d	17.1 ± 0.2^a^
Male 58d	24.1 ± 0.7^b^
Male 70d	24.9 ± 0.5^b^
Male 98d	28.2 ± 0.5^c^
Female 30d	15.7 ± 0.7^a^
Female 58d	19.1 ± 0.6^b^
Female 70d	21.8 ± 0.4^c^
Female 98d	24.1 ± 0.5^d^

*Numbers that share a letter within an experiment are not significantly different from one another (*P* < 0.05)*.

#### Experiment 2

In females, there was also a significant change in body weight throughout adolescence and young adulthood (*F*_(3,16)_ = 38.92, *P* < 0.05), such that body weight significantly increased in a linear manner at all four ages measured (Table 1). For the measurements made in the hippocampus, there were both significant increases in the cross-sectional area of the dentate gyrus and decreases in the number of Ki-67-positive cells across the ages (*F*_(3,16)_ = 4.52 and 23.52. respectively, *P* < 0.05). For cross-sectional area, the dentate gyrus was significantly larger in 98-day females compared to 30-day, 58-day, or 70-day females (Figure 2C), while the Ki-67 cells per 100 μm^2^ of dentate gyrus show the same adolescent-related decline as males, with 30-day females having the greatest number of Ki-67 cells compared to all the other ages (Figure 2D). Also similar to the males, it appears Ki-67 cell number continues to decline in the dentate gyrus through late adolescence and young adulthood, with the lowest number of cells in the 98-day females.

### Experiments 3 and 4: Hippocampal Cellular Proliferation and Number of Immature Neurons Following Oral Corticosterone Treatment During Adolescence or Adulthood

#### Experiment 3

**Figure 3:**
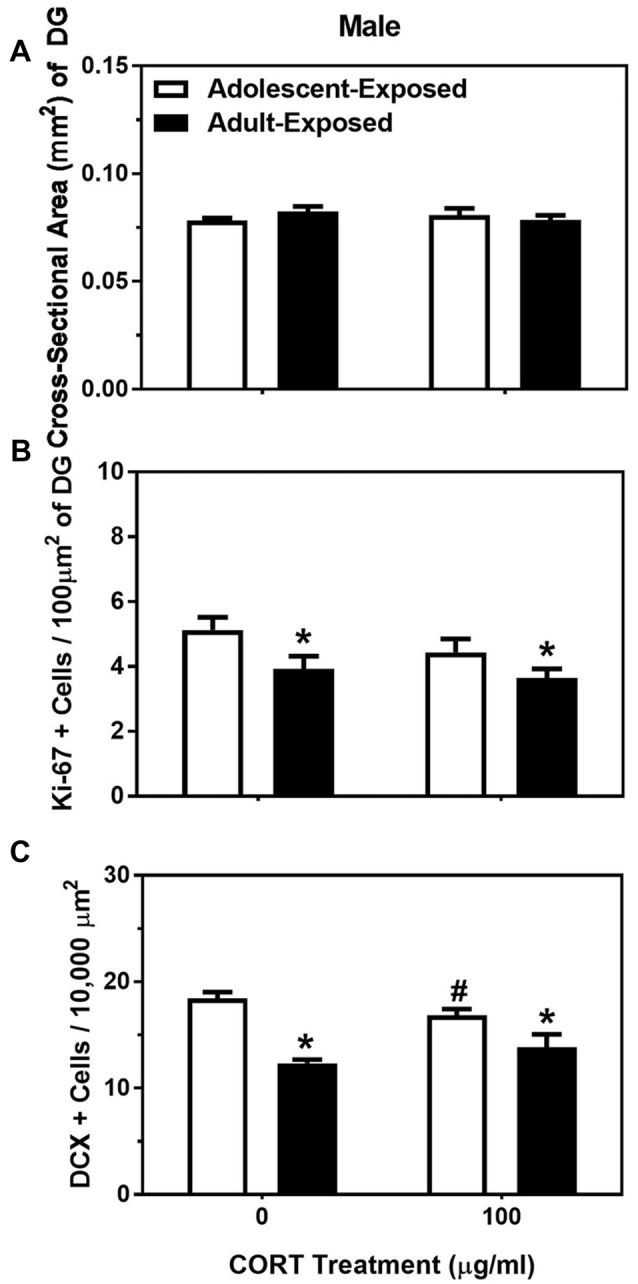
Mean (±SEM) cross-sectional area (mm^2^) of the DG **(A)**, number of Ki-67-positive cells per 100 μm^2^ of DG **(B)** and number of DCX-positive cells per 10,000 μm^2^ **(C)** in male mice exposed to either 0 or 100 μg/ml of corticosterone (CORT) during adolescence (white bars) or adulthood (black bars). Asterisks indicate a significant difference between the adolescent-exposed and adult-exposed animals, while ^#^indicates a significant difference between the 0 μg/ml and 100 μg/ml dose of CORT in the adolescent-exposed animals.

For body weight in the adolescent- and adult-treatment males, main effects were found, such that adult-treated males weighed more than adolescent-treated males, and animals treated with 100 μg/ml of corticosterone were heavier than the animals treated with 0 μg/ml of corticosterone (*F*_(1,28)_ = 75.35 and 41.12, respectively, *P* < 0.05; Table 2). For the dependent variables measured in the dentate gyrus, we found no main effects or interaction of age of exposure or treatment condition on the cross-sectional area (Figure 3A), and only a significant main effect of age on the number of Ki-67 cells per 100 μm^2^ of the dentate gyrus (*F*_(1,24)_ = 7.17, *P* < 0.05). Specifically, the 58-day animals treated with either 0 or 100 μg/ml of corticosterone during adolescence had a greater number of Ki-67 cells than the 98-day animals treated with either 0 or 100 μg/ml of corticosterone during adulthood (Figure 3B). There was no main effect of corticosterone treatment or interaction between age of exposure and corticosterone treatment on Ki-67 cells number per 100 μm^2^ of dentate gyrus.

**Table 2:** Mean (±SEM) body weight of male and female mice in Experiments 3 (male mice) and 4 (female mice) treated with either 0 or 100 μg/ml of corticosterone (CORT) during either adolescence (30–58 days) or young adulthood (70–98 days).

Sex and age of exposure	Treatment condition	Body weight (g)
Male Adolescent-Exposed	0 μg/ml CORT	23.6 ± 0.5
Male Adolescent-Exposed	100 μg/ml CORT	26.3 ± 0.8*
Male Adult-Exposed	0 μg/ml CORT	27.7 ± 0.5
Male Adult-Exposed	100 μg/ml CORT	32.8 ± 0.5*
Female Adolescent-Exposed	0 μg/ml CORT	20.6 ± 0.4
Female Adolescent-Exposed	100 μg/ml CORT	24.4 ± 0.9*
Female Adult-Exposed	0 μg/ml CORT	23.4 ± 0.6
Female Adult-Exposed	100 μg/ml CORT	27.8 ± 1.0*

*Asterisks indicate a significant difference between the 0 μg/ml and 100 μg/ml treatment conditions. Note that the significant main effects for age of exposure in Experiments 3 and 4 are not indicated*.

For the number of DCX-positive cells in the dentate gyrus, we found both a significant main effect of age of exposure as well as a significant interaction between age of exposure and corticosterone treatment (*F*_(1,24)_ = 36.35 and 4.35, respectively, *P* < 0.05). Specifically, like the number of Ki-67-positive cells, the number of DCX cells were greater in the 58-day compared to 98-day males, independent of treatment. For the interaction, we found a slight, but significant suppressive effect of corticosterone on DCX cell number, but only when the exposure occurred during adolescence (Figure 3C). There was no significant main effect of treatment condition on the number of DCX cells in the dentate gyrus of males.

#### Experiment 4

**Figure 4:**
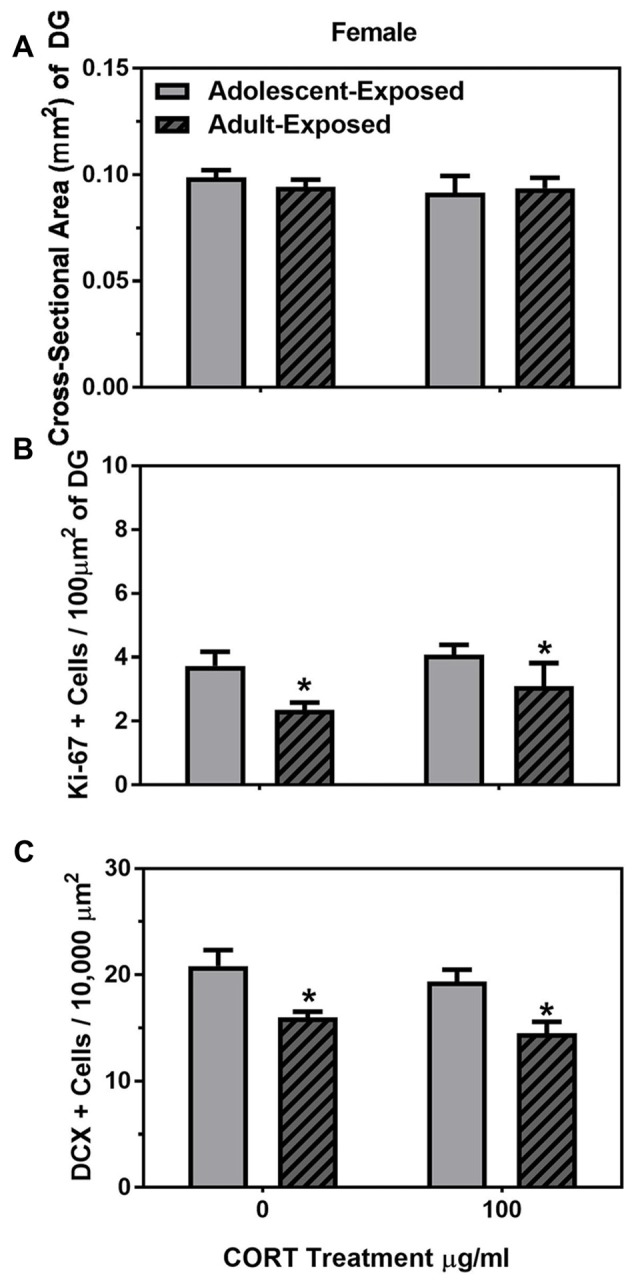
Mean (±SEM) cross-sectional area (mm^2^) of the DG **(A)** number of Ki-67-positive cells per 100 μm^2^ of DG **(B)** and number of DCX-positive cells per 10,000 μm^2^ **(C)** in female mice exposed to either 0 or 100 μg/ml of CORT during adolescence (light gray bars) or adulthood (dark gray bars with hash marks). Asterisks indicate a significant difference between the adolescent-exposed and adult-exposed animals.

Similar to males, main effects were found on the body weights of the adolescent- and adult-treatment females, such that adult-treated females weighed more than adolescent-treated females, and females treated with 100 μg/ml of corticosterone were heavier than the females treated with 0 μg/ml of corticosterone (*F*_(1,26)_ = 14.16 and 25.28, respectively, *P* < 0.05; Table 2). Also similar to the males, we found no main effects or interaction of age of exposure or treatment condition on the cross-sectional area of the dentate gyrus (Figure 4A), but found a significant main effect of age on the number of Ki-67 cells per 100 μm^2^ of the dentate gyrus (*F*_(1,22)_ = 6.03, *P* < 0.05). Like the males, 58-day females treated with either 0 or 100 μg/ml of corticosterone during adolescence had a greater number of Ki-67 cells than 98-day females treated with either 0 or 100 μg/ml of corticosterone during adulthood (Figure 4B). There was no main effect of corticosterone treatment or interaction between age of exposure and corticosterone treatment in the females.

For DCX cells counts, we found only a significant main effect of age of exposure (*F*_(1,22)_ = 20.81, *P* < 0.05), such that 58-day females had a greater number of DCX-positive cells than 98-day females, independent of treatment condition (Figure 4C). There was no main effect of treatment condition or interaction between age of exposure and treatment condition on the number of DCX-positive cells in the female dentate gyrus.

## Discussion

These data indicate that cellular proliferation in the dentate gyrus showed significant declines during adolescent development in both male and female C57BL/6N mice. Furthermore, despite significant somatic changes in response to these corticosterone treatments, we found little effect of these treatments on hippocampal proliferation and the number of immature neurons. Specifically, chronic corticosterone exposure had no effect on these parameters in adolescent- or adult-treated females, and in males, only the number of immature neurons was affected when these treatments occurred during adolescence. Thus, counter to the original hypothesis, these data indicate that the substantial change in hippocampal proliferation and neurogenesis that occurs during adolescence is largely resistant to these chronic oral corticosterone treatments.

The metabolic function of the animals was influenced by this exposure to corticosterone, as has been reported previously (Karatsoreos et al., 2010; Cassano et al., 2012; Kinlein et al., 2017). That is, these corticosterone treatments led to significant weight gain in the adolescent- and adult-exposed subjects. Thus, the relative lack of corticosterone-induced changes in cellular proliferation and number of immature neurons indicates a dissociation between the effects of corticosterone on somatic and neurobiological functions. It is possible that a higher dose of corticosterone would have yielded a greater effect on the neurobiological parameters we assessed, as the slight decrease in DCX cell number in the adolescent-treated males suggests that the 100 μg/ml dose of corticosterone might be near an effective threshold. Furthermore, a longer time of exposure might be needed, as others have reported that 7 weeks of oral corticosterone exposure can reduce hippocampal proliferation in adult male mice (David et al., 2009). Regardless, if this method of delivery is to be used to understand the influence of corticosterone on either hippocampal cellular proliferation or neurogenesis in either adolescent or adult mice, then additional experiments will be needed to address these dose response and time course issues.

Given the relative absence of an effect of corticosterone on hippocampal proliferation in the present study, these data however do raise an interesting possibility that the metabolic changes induced by these treatments might have protected the dentate gyrus from any adverse effects of chronic corticosterone exposure. For example, previous research has indicated that metabolic hormones, such as leptin, can be neuroprotective (Avraham et al., 2011), and leptin has been shown to reverse the suppressive effects of chronic unpredictable stress on hippocampal neurogenesis in rats (Garza et al., 2012). Leptin levels have been reported to be increased in mice treated with the dose of oral corticosterone used in the present study (Karatsoreos et al., 2010). Moreover, the dentate gyrus has a relatively high expression level of leptin receptors in C57BL/6 mice (Huang et al., 1996). Thus, it is possible that the elevated leptin levels induced by these treatments might have mitigated the suppressive effects of corticosterone on hippocampal cellular proliferation. Future experiments will be needed to address this possibility.

We found that, independent of corticosterone treatment, cell proliferation and the number of immature neurons were significantly different between 58 days and 98 days of age in both females and males, indicating that these parameters of plasticity are not static during adulthood, but continue to decrease. While previous work has observed a substantial decrease in hippocampal cellular proliferation and neurogenesis between adolescent and adult mice (He and Crews, 2007), differences have not been previously measured at different ages during young adulthood in mice. Given the important role of hippocampal proliferation in neurobehavioral functions, ranging from learning and memory to emotionality (Bannerman et al., 2014), future studies will need to probe the functional implications of these changes in the dentate gyrus during both adolescence and young adulthood.

Taken together, these data indicate that despite profound changes in hippocampal cellular proliferation and neurogenesis during adolescence and adulthood, chronic oral corticosterone exposure was largely unable to disrupt this developmental process in male or female mice. Though oral corticosterone may serve as a useful model to understand both adolescent- and adult-related differences in metabolic dysfunctions (Kinlein et al., 2017), the present data suggest this method may not be an effective way to examine the role of corticosterone on hippocampal neurogenesis in mice. Instead, we propose that this methodology may be appropriate for future studies trying to understand the interaction between metabolic dysregulation and neurobiological functions, and the potential compensatory mechanisms that metabolic hormones may have on deleterious effects of chronic exposure to stress or stress-related hormones on the brain and behavior.

## Author Contributions

AShome and RR designed and conducted the experiments, while RS and ASiddiqui helped conduct the experiments. All authors contributed to the writing and editing of the manuscript.

## Conflict of Interest Statement

The authors declare that the research was conducted in the absence of any commercial or financial relationships that could be construed as a potential conflict of interest.
